# Event and Comment

**Published:** 1921-06

**Authors:** 


### DENTAL REGISTER

THE MAGAZINE OF DENTISTRY

Vol. LXXV JUNE, 1921 No. 9

### EVENT AND COMMENT

**The Danger of Extremes in Dental Practice**

In the practice of a profession, especially one so new as dentistry, there are many chances for us to dogmatize and follow, unknowingly, erroneous methods or choose wrong conclusions rather than those well considered or wisely determined. It is very necessary that we should choose proper methods, or systems of well thought out procedures, in making a diagnosis that is sound in theory and will harmonize with good methods of practice. There seems to be at this time a tendency to adopt many radical and fadish notions of practice, as regards diseased teeth. Most of these fancies have not yet been approved conclusively, and naturally we, in many instances, do not know what to say or do when undertaking active practice. At least we have not had the timely advice of expert testimony, and while we may believe in the logic of our faith, we have no proved grounds for absolute dogmatism. There are so many unknown items in our procedures that we do not know much about for certain, and we can not know more until we have more scientific bases for action.

Physicians are supposed to be thoroughly grounded in medical sciences, and still they are in much doubt as to most matters of practice, and have to allow rather free use of their reasoning and history of practical experience in making up a report of a clinical finding. They tell us to extract every questionable tooth, for fear of infecting a vital tissue of the general system; this, regardless of a possible injury to some valued function of the dental organs. There may be sufficient reason for much of the systemic injuries from dental diseases, but it does seem that it will be a mistake to insist on radical treatments when the causes are not clearly made out. Present dental practice is well intrenched in safe and good dental procedures, from scientific studies, as well as ripe experiences; and we must be shown before we make radical changes in present methods. We expect by study and experience we shall, by and by, know the reasons why teeth decay, and their soft structures become pathologic, and then we shall know how to correct the mistakes we have made in diagnosis and therapeutics. But it does not seem fair to attach so much blame until we are more sure of our facts and the bearings these facts have in relation to all curative measures are working together in co-operation. It seems logical to believe that all the facts are needed and when this is done we shall work together more desirably. At any rate, there is no use in radical action when there is a chance for making a wrong action because we do not know the whole truth which could be made clear by time and experience. In other words, don't permit, or precipitate, a stampede until we are more certain of the facts. We certainly need to keep up our courage and strive to know the best practical measures to use before we permit an irreparable injury. We can at least minimize all local pathologic influences by prevention and treatment so far as may be practicable.

---

**Honor to Dr. Charles N. Johnson** A large attendance of the members of the profession assembled in the La Salle Hotel, Chicago, April 11, 1921, to commemorate his forty years of the practice of dentistry. Nearly a thousand people attended and the program was most interesting and attractive. Dr. Johnson began the study and practice of dentistry in Callingwood, Ontario, Canada, but after three years removed to Chicago, where he has lived ever since.

He is one of the most earnest and devoted practitioners that we have ever known. The profession has done wisely to extend to him this gracious honor, and no man has more faithfully devoted himself to the dental profession. As a practitioner he has done a tremendous service for the public; as a teacher in the college he has similarly distinguished himself and endeared himself to thousands of students; as a writer of books, papers and magazines he has probably contributed more separate literary articles than any other dentist; and as a genial friend and co-operator in all dental services he has simply been indefaticable. How one man could ever do so much that is really worth while doing is beyond our conception. He had a big brain; a fine inheritance from Godly parents, who trained him early to strictest probity of character; and nature seemed to have most generously endowed him with all the other fine ideals of character which he and his multitude of friends gathered about him on every occasion.

He has the peculiar and delightful charm which makes one feel that he is his one best friend. He deserves the encomiums of his friends. Our trials and troubles would soon leave us if we had more of the spirit of Johnson in our hearts.

The following poem by Dr. Johnson is characteristic of his spirit and life:

MY HOUR

There's an hour not recorded by the clocks of standard time,
Not remembrancer of summer nor a hint of winter's rime,
It's an hour quite creative—by a myriad fancies fed,
When the embers slowly smoulder, and the folks are all in bed.

Then I'm king in my dominion, with a little desk my throne,
Swarmed about by countless subjects, though I seem to sit alone;
And I hold such sweet communion with the play-folk of my head,
When the night winds softly murmur and the real folk are in bed.

Nothing but a pen and paper, yet a world lies at my feet,
Waiting only touch of magic to portray the scene complete;
Visions weird and visions wondrous, like a message from the dead,
Stealing softly through and through me, while the dear ones are
in bed.

Harmless ghosts and little goblins—children of another clime,
Some sedate and others sportive—others lowly, some sublime;
All are weaving with a fabric fine as any silken thread,
Tales and legends of the elf-land while the folks are all in bed.

Time is nothing but a shadow, space is spanned by winged thought;
All the myriad things of daytime now appear as things of naught.
All the real and all the unreal, thus mysteriously wed
By this mystic ceremony, while the folks are all in bed.

Thus I sit in waking dreamland as the moments steal away,
Quite unconscious, while the clock is striking in another day.
What though half I think's unwritten, and the other half unread,
Priceless is this cherished hour when the folks are all in bed.

—*Charles Nelson Johnson.*

